# Interactions of Epinephrine and Norepinephrine with Intralipid Emulsion Studied by Capillary Electrokinetic Chromatography

**DOI:** 10.1002/jssc.70188

**Published:** 2025-06-09

**Authors:** Dumidu Perera, Henri K. M. Ravald, Veronika Šolínová, Václav Kašička, Ju‐Tae Sohn, Susanne K. Wiedmer

**Affiliations:** ^1^ Department of Chemistry University of Helsinki Helsinki Finland; ^2^ Institute of Organic Chemistry and Biochemistry Czech Academy of Sciences Prague Czech Republic; ^3^ Institute of Medical Science Gyeongsang National University Jinju‐si Gyeongsangnam‐do Republic of Korea; ^4^ Department of Anesthesiology and Pain Medicine Gyeongsang National University College of Medicine Gyeongsang National University Hospital Jinju‐si Gyeongsangnam‐do Republic of Korea

**Keywords:** binding constant, distribution coefficient, electrokinetic chromatography, Intralipid, lipid emulsion

## Abstract

Epinephrine and norepinephrine are widely used to treat cardiovascular collapse during resuscitation with lipid emulsions in the treatment of drug toxicity including local or non‐local anesthetics. The effect of the lipid emulsion Intralipid on the vasoconstriction induced by epinephrine or norepinephrine is, however, still unknown. In this study, the interaction of epinephrine and norepinephrine with the intravenous Intralipid emulsion was investigated by capillary electromigration techniques. Capillary electrokinetic chromatography was performed to determine the distribution coefficients by running the analytes under different experimental conditions (temperature, ionic strength, and pH) through a capillary filled with the background electrolyte containing Intralipid emulsion. In addition, the binding constants of the epinephrine and norepinephrine complexes with Intralipid emulsion were determined based on the effective electrophoretic mobility data obtained by electrokinetic chromatography at a wide concentration range of Intralipid emulsion. The obtained binding constants, as well as the distribution coefficients determined by electrokinetic chromatography, confirm that epinephrine and norepinephrine are hydrophilic compounds and that they are minimally distributed into the Intralipid emulsion. Therefore, their application as drugs for vasoconstriction upon Intralipid emulsion treatment is well motivated.

**Abbreviations**
CAC: critical aggregation concentration
DMSO: dimethyl sulfoxide
EKC: electrokinetic chromatography
ILE: Intralipid emulsion
LE: lipid emulsion
PBS: phosphate buffered saline

## 1 Introduction

Intravenous lipid emulsions (LEs) are aqueous suspensions of lipid droplets, that originally were developed to provide parenteral nutrition to critically ill patients or to persons who are unable to obtain adequate external nutrition. Different brands of LEs are commercially available, such as Intralipid, which is composed of soybean oil (10%, 20%, or 30% m/v), egg yolk phospholipids (1.2% m/v), glycerin (2.25% m/v), and water. Beyond nutrition, LEs have been investigated for their potential in treating drug toxicity. Research began with Weinberg et al. who observed that Intralipid emulsion (ILE) infusion reduced bupivacaine‐associated cardiotoxicity in rats, likely via a lipid sink mechanism [1]. Xanthos et al. demonstrated that ILE could mitigate hypotensive effects in pigs overdosed with amiodarone [2]. Harvey and Cave highlighted ILE's superiority over sodium bicarbonate in reversing clomipramine toxicity in rabbits [3]. In 2006, the first successful use of a LE to resuscitate a patient from a prolonged cardiac arrest due local anesthetic (bupivacaine) toxicity was reported [4]. ILE was used in that work. In a recent review, it was suggested that the “dynamic lipid shuttle (subway)” is currently a widely accepted mechanism [5]. According to that, the LE creates a multitude of lipid aggregates in the blood, and the lipid phase of the LE subsequently absorbs drugs with high lipophilicity from areas of high blood supply followed by the transportation of that to the liver, muscle, and adipose tissue for detoxification and storage. Barker et al. conducted an in vitro study, and they observed that 20 out of the 27 studied drugs, reduced in concentration when ILE was present in the plasma [6].

**FIGURE 1:**
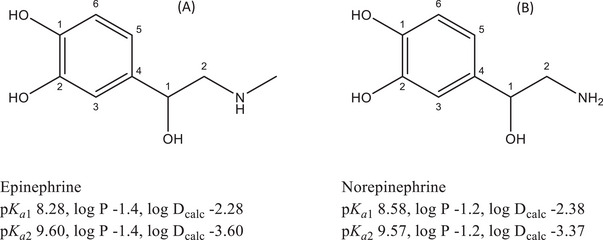
Chemical structures and the corresponding p*K*_a_, log *P*, and log *D*_calc_ values of epinephrine (A) and norepinephrine (B). The p*K*_a1_ (amino group) values were literature values [9]. The p*K*_a2_ (one of the phenolic groups) values were calculated using Advanced Chemistry Development (ACD/Labs) Software V11.02 and the log *p* values were obtained using XLogP3 3.0 Software. The log *D*_calc_ values were calculated using Equation (5).

Epinephrine and norepinephrine (also called adrenaline and noradrenaline, respectively) are stress hormones and neurotransmitters produced by the adrenal gland and function as part of the “fight or flight” response. The chemical structure of epinephrine consists of a benzene ring with secondary hydroxy alkyl and secondary amino alkyl groups and two phenolic hydroxyl groups making it a catecholamine (Figure 1). Given its stability and reactivity, epinephrine is sensitive to heat, light, air, and alkalis, and metals like copper, iron, and zinc can cease its activity [7]. Epinephrine is known to be soluble in water mainly due to its above secondary hydroxy alkyl and alkyl amino groups and the two hydroxyl (phenolic) groups. Its solubility depends on the pH and is the lowest at pH 9.4 [8]. Epinephrine is amphoteric compound having a p*K*_a1_ value of the secondary alkylamino group equal to 8.28 [9] and a predicted p*K*_a2_ value of the first phenolic group equal to 9.60 ± 0.10 (calculated using Advanced Chemistry Development [ACD]/Labs Software V11.02). It makes epinephrine almost 90% positively charged at pH 7.4, that is the pH value at which the most experiments in this work were conducted.

Norepinephrine is also a catecholamine having the same catechol groups as epinephrine [7], as shown in Figure 1. The only structural difference as compared to epinephrine is in the end amino group, which is a primary amine. Other than the structural similarity, norepinephrine shows similar stability as well. Norepinephrine is highly soluble in alkali and diluted hydrochloric acid solutions and quite soluble in water, ethanol, and diethyl ether [10]. This compound is also an amphoteric compound having a p*K*_a1_ value of 8.58 for the primary amino group [11] and a predicted p*K*_a2_ value of 9.57 ± 0.10 for the first phenolic group (calculated using Advanced Chemistry Development [ACD]/Labs Software V11.02). It means that norepinephrine is a more than 90% positively charged compound at pH 7.4, at which most of the experiments were conducted.

Epinephrine is used as a medical treatment for conditions such as anaphylaxis and cardiac arrest, while norepinephrine is used to treat dangerously low blood pressure. Epinephrine's effect in resuscitation along with LE administration has been previously researched in in vivo models. Siqueira et al. studied the effect of ILE and epinephrine (alone or in combination) on the resuscitation of piglets under local anesthetic‐induced cardiac toxicity and found similar results for all options; however, epinephrine was found to increase electrocardiogram abnormalities after resuscitation, alone and combined with ILE [12]. Mauch et al. also resuscitated piglets with epinephrine and ILE, following bupivacaine‐induced intoxication, noting that only epinephrine (alone or with LE) restored spontaneous circulation and LE showed stabilizing effects thereafter [13]. In another study, the efficacy of ILE and Lipovenoes LE administration to epinephrine‐based resuscitation of asphyxia‐induced cardiac arrest in an in vivo model of rats was investigated [14]. The addition of LEs were found to improve the resuscitation success compared to epinephrine alone. Harvey et al. studied the role of epinephrine in ILE‐based resuscitation of toxin‐induced cardiac arrest using a rabbit model [15]. Epinephrine improved the return of spontaneous circulation compared to saline/ILE control but was subsequently associated with declining hemodynamic variables. Karcioglu et al. found that combining ILE and epinephrine improved survival rates in rabbits with levobupivacaine‐induced cardiac arrest compared to saline/epinephrine [16]. The study by Fellows et al. assessed ILE's impact on plasma epinephrine and norepinephrine levels in six healthy men, discovering no significant changes in concentration, 60 min post‐administration [17].

Moreover, in vitro research has been conducted on LE interactions with pharmaceutical drugs. French et al. determined whether the extraction of various drugs with ILE can be predicted by specific drug properties [18]. The drugs investigated along with Intralipid were added individually to human drug‐free serum, which was shaken and then incubated at 37°C. They found that the ability of a drug to bind to the LE was dependent on the drug's partition coefficient. In addition, the prediction of binding was improved by combining the partition coefficient with the volume of distribution.

The estimation of drug solution‐membrane partitioning of hydrophilic drugs by, for example, immobilized artificial membrane (IAM) chromatography, also named biomimetic chromatography, often shows big variation in the partitioning values, as compared to octanol/water partitioning. The commercial IAM chromatography phases are hampered by the need for method validation for every small variation in the conditions and the lack of a larger variety of commercially available reproducible stationary phases [19]. Recently, a novel methodology for estimating liposome/water partition coefficients was introduced [20]. The methodology, which was much based on dialysis through a semipermeable membrane, gave partition coefficients for the analyzed compounds comparable to data obtained by differential pulse voltammetry, HPLC‐UV, and GC‐MS (after derivatization of the compounds).

We have previously conducted research on LE‐drug interactions using ILE and liposomes independently as pseudostationary phases (PSPs) in capillary electrokinetic chromatography (EKC) [21, 22, 23, 24]. Muhonen et al. developed a method for studying the mechanism between the local anesthetics bupivacaine, lidocaine, and prilocaine and ILE and other synthetic lipid dispersions [22]. In a follow up study, Laine et al. studied further the interactions of immobilized ILE with drugs by LC‐MS [21]. In that study we observed that the interaction was stronger when the ILE concentration was increased, and that ILE interacted most strongly with the most lipophilic drug, bupivacaine. Ravald et al. determined the binding constants of complexes of β‐blockers and ILE using EKC [23]. The binding constants strongly related to the distribution coefficient log *D* values of the β‐blockers, with more lipophilic β‐blockers interacting more strongly with the ILE.

There are some concerns about whether LEs can absorb vasopressors, such as epinephrine and norepinephrine, used to treat cardiovascular collapse during LE resuscitation due to drug toxicity. Therefore, the purpose of this study was to determine by EKC whether ILE has any interactions with the hydrophilic drugs epinephrine and norepinephrine, which could potentially influence resuscitation situations involving both the administration of ILE and the compounds. For assessing these interactions, the distribution coefficients and the binding constants of epinephrine and norepinephrine complexes with ILE, were experimentally determined by EKC. The impact of the temperature, ionic strength, and pH on the interactions were studied utilizing EKC at two different temperatures, three different ionic strengths, and six different pH values. The data shown below evidences negligible binding between the drugs and the ILE.

## 2 Experimental

### 2.1 Chemicals and Buffer Solutions

ILE at 200 mg/mL concentration (20% m/v) was obtained from Fresenius Kabi, Sweden (Vnr 194548). The buffer reagents disodium hydrogen phosphate dodecahydrate (Na_2_HPO_4_·12 H_2_O) and sodium dihydrogen phosphate monohydrate (NaH_2_PO_4_·H_2_O) were from J.T. Baker (Deventer, the Netherlands) and Merck (Darmstadt, Germany), respectively. HPLC grade 99.5% dimethyl sulfoxide (DMSO) was from Labscan (Dublin, Ireland). LC‐MS grade ≥ 99.9% methanol from Fisher Chemical (Geel, Belgium) was used as solvent for the analyte stock solutions. The standard pH solutions (4.0, 7.0, and 10.0) used for pH meter calibration were purchased from Merck (Darmstadt, Germany).

### 2.2 Buffer and Stock Solution Preparation

Appropriate amounts of Na_2_HPO_4_·12 H_2_O and NaH_2_PO_4_·H_2_O (constituents of the phosphate buffered saline (PBS) used as a BGE) were calculated for each relevant pH and ionic strength. The required amounts were weighted using a Sartorius BP301S analytical balance and dissolved in Milli‐Q water, obtained from a water purification system Milli‐Q Direct‐Q 3 UV. WTW InoLab pH 7110 pH meter was used for measuring the pH and before the analysis, solutions were filtered through a 0.45 µm membrane filter. The stock solutions of epinephrine and norepinephrine samples were initially prepared as 1 mg/mL in methanol and water, respectively, and then diluted using PBS to the required concentrations. For EKC studies, the 20% m/v (200 mg/mL) ILE solution was diluted to 1% m/v solutions using PBS with relevant ionic strength and pH for each studied parameter. Samples were run in BGE with 1% m/v ILE to obtain the relevant migration times required for calculations of effective electrophoretic mobilities from the EKC experiments.

### 2.3 Capillary Electromigration Methods

An Agilent Technologies 7100 CE capillary electrophoresis system equipped with a spectrophotometric UV‐absorption diode array detector was used. The capillary was a bare fused silica capillary with outer polyimide coating (Polymicro Technologies) with a total length of 68.5 cm and a length of 60 cm to the detector. The capillary inner and outer diameters were 50 and 360 µm, respectively. The software was Agilent OpenLAB CDS ChemStation Edition c.01.05.

Throughout the experiments, the capillary was flushed with 0.1 M NaOH for 3 min, with H_2_O for 2 min, and with PBS for 5 min at high pressure (ca 940 mbar) before a new type of BGE was taken into use (i.e., when the ILE concentration, BGE pH, or BGE ionic strength was changed). Before each injection, the capillary was flushed with BGE for 1 min. All the samples were injected at 100 mbar pressure at 10 s. Migration times were recorded, and all runs were repeated at least three times. A solution of 0.05% v/v DMSO in water was used as the electroosmotic flow (EOF) marker. The applied separation voltage was 30 kV. The UV spectra of the runs were recorded at the wavelengths 190–400 nm with 2 nm steps and the spectra of the analytes were saved in the UV spectrum library. Electropherograms were extracted at wavelengths of 200, 214, 230, and 254 nm. The temperature was 25°C or 37°C. The studied ionic strengths were 20, 50, and 100 mM and the pH values were in the range of 6.8–7.8.

### 2.4 Determination of the Retention Factors and the Distribution Coefficients

Both in capillary electrophoresis (CE) (alternatively also called capillary zone electrophoresis [CZE]) and EKC, the effective electrophoretic mobilities of epinephrine and norepinephrine, *µ*_eff_, were calculated using the Equation (1):

(1) $$\mu_{\mathrm{eff}}=\frac{L_{\mathrm{tot}}L_{\mathrm{eff}}}{U_{\mathrm{sep}}}\left(\frac{1}{t_{\mathrm{mig}}}-\frac{1}{t_{\mathrm{eof}}}\right)$$

where *L*_tot_ and *L*_eff_ are the total and the effective (to the detector) capillary lengths, respectively, *U*_sep_ is the separation voltage, *t*_mig_ and *t*_eof_ are the migration times of the analyte and of the EOF marker (DMSO), respectively. The effective mobility of ILE (mobility of the ILE PSP, *µ*_psp_) was obtained by injecting ILE as a sample (analyte) and performing CE runs with PBS as the BGE at each of above‐mentioned conditions and using Equation (1) for the mobility calculation. For calculations of the binding constants of the drug‐ILE complexes, EKC experiments were conducted at 13 different ILE concentrations in the range of 0%–8.35% m/v (corresponding to 0–533 mM). All runs were repeated at least three times.

The obtained mobilities were used to calculate the retention factors (*k*) of the analytes using Equation (2),

(2) $$k=\frac{\mu_{EKC}-\mu_{0}}{\mu_{psp}-\mu_{EKC}}$$

where *µ*_EKC_ is the electrophoretic mobility of the analyte under EKC conditions, *µ*_0_ is the electrophoretic mobility of the analyte under conventional CE conditions, that is using a BGE free of ILE, and *µ*_psp_ is the electrophoretic mobility of the pseudo stationary phase [25]. Subsequently, the distribution coefficient of the analytes, *D*_lip_, (the ratio of the concentrations of the sum of both noncharged and charged forms of analytes in the lipid and aqueous phases) was obtained as a ratio of their retention factor *k* and the phase ratio *ϕ* according to Equation (3) [26]:

(3) $$D_{lip}=\frac{k}{\phi }$$

The phase ratio, *ϕ*, was calculated using the Equation (4) [26]:

(4) $$\phi =\frac{V_{lip}}{V_{aq}}=\frac{V_{spec,vol}\times M\times \left(c_{lip}-CAC\right)}{1-\left(V_{spec,vol}\times M\times \left(c_{lip}-CAC\right)\right)}$$

where *V*_lip_ is the volume of the lipid phase, *V*_aq_ is the volume of the aqueous phase, *V*_spec,vol_ is the partial specific volume of lipids (0.996 mL/g at 30°C [27]), *M* is the average lipid molar mass, *c*_lip_ is the total concentration of lipids and surfactants, and *CAC* is the critical aggregation concentration. The *CAC* value is several orders smaller than the other concentrations used and was thus neglected [28]. The phase ratio for the used 1% m/v ILE dispersion was estimated to be equal to 1.27 × 10^−2^ based on the equation above.

The calculated distribution coefficients (log *D*_calc_) were determined using the Equation (5) [29]:

(5) $$\log D_{\mathrm{calc}}=\log P-pK_{a}+\mathrm{pH}$$

where *P* is the partition coefficient, and p*K*_a_ is the acid dissociation constant (the values of *P* and p*K*_a_ of epinephrine and norepinephrine are presented in legend of Figure 1).

### 2.5 Determination of the Binding Constants

The strength of interactions between the well‐defined individual molecules of epinephrine and norepinephrine and the complex composition of the ILE‐based PSP in EKC was investigated using the simplifying assumption that ILE PSP is taken as a “monomolecular” PSP or a receptor R with an average molar concentration, *c*_R_. After this assumption it was possible to evaluate the interactions between epinephrine or norepinephrine (applied as analytes A) and the receptor R (ILE dispersed homogeneously in the BGE inside the whole capillary) by the binding constant, *K*_b_, of the analyte‐receptor (AR) complex using the following theory.

The interaction between analyte A (epinephrine or norepinephrine) and receptor R (ILE) with 1:1 stoichiometry is described by the following equilibrium:

(6) A+R⇄AR

and characterized by the apparent binding constant, *K*_b_ (further called only as the binding constant), defined by Equation (7):

(7) $$K_{b}=\frac{c_{\mathrm{AR}}}{c_{A}c_{R}}$$

where *c*_A_, *c*_R_, and *c*_AR_ are the equilibrium concentrations of the analyte A, receptor R, and complex AR, respectively. With respect to relatively high concentrations of the receptor R in the BGE, the equilibrium concentration of receptor R, *c*_R_, is taken as equal to the total concentration of the receptor R in the BGE.

The effective mobility of the analyte A, *µ*_eff,A_, in the BGE containing receptor R is defined as a sum of the products of (i) electrophoretic mobilities of its non‐complexed (free) and complexed forms and (ii) molar fractions of these forms related to the all forms of the analyte A:

(8) $$\mu_{\mathrm{eff},A}=\mu_{A}\frac{c_{A}}{c_{A}+c_{\mathrm{AR}}}+\mu_{\mathrm{AR}}\frac{c_{\mathrm{AR}}}{c_{A}+c_{\mathrm{AR}}}$$

where *µ*_A_ and *µ*_AR_ are the actual electrophoretic mobilities of the free analyte A and the complex AR, respectively.

Combination of Equations (7) and (8) gives the following equation for the effective mobility of the analyte A, *µ*_eff,A_:

(9) $$\mu_{\mathrm{eff},A}=\frac{\mu_{A}+\mu_{\mathrm{AR}}K_{b}c_{R}}{1+K_{b}c_{R}}$$

Equation (9) describes the dependence of the effective mobility of analyte A on the concentration of the receptor R in the BGE. In this equation, the electrophoretic mobility of analyte A, *µ*_A_, is equal to its mobility in the BGE free of receptor R determined by CE (CZE), and the binding constant, *K*_b_, and the electrophoretic mobility of the AR complex, *µ*_AR_, are the parameters of this equation. They can be obtained by nonlinear regression analysis of this dependence using Equation (9) as the fitting function [30].

## 3 Results and Discussion

The distribution of epinephrine and norepinephrine into ILE was investigated through determining the distribution coefficients log *D* of the drugs in ILE and the binding constants *K*_b_ of complexes of the drugs with ILE using EKC. The impact of the ionic strength, the temperature, and the pH on the distribution was investigated by utilizing three different ionic strengths, two different temperatures, and six different pH values.

### 3.1 Determination of the Distribution Coefficients

In our previous study, traditional liquid–liquid extraction (LLE) was carried out to determine the compound distribution in ILE [31]. The technique is time‐ and labor‐consuming and requires several analytical steps (extraction, centrifugation, phase separation, and quantitative analysis). In addition, LLE is rather unselective, and separation of the phases can sometimes be difficult. Temperature fluctuations, if not carefully controlled, can also affect the distribution of the compounds between the phases. In our previous work, the extractions were only done at room temperature and at pH 7.4, however, at different ionic strengths. The very low log *D*_lip_ values obtained suggest that ILE did not significantly alter vasoconstriction induces by epinephrine or norepinephrine. However, to get more insight into the interactions, studies were done here using a much more straightforward technique, namely EKC.

During intravenous drug dosing, the drugs are typically solubilized in solutions with rather high ionic strength (e.g. Krebs solution with an ionic strength of ca 158 mM). Therefore, the research question here was whether the concentration of the buffer solution, a change in the temperature, or the pH value would affect the distribution of the compounds into ILE. Three different ionic strengths of the buffers, that is, 20, 50, and 100 mM, were investigated. The negative log *P* values (the ratio of concentrations of noncharged forms of analytes in organic and aqueous phases) and calculated log *D*_calc_ values of the compounds, shown in Figure 1, suggest that the compounds are highly hydrophilic.

In our previous study [31], the quantification of the compounds in the phases after LLE was done by CZE. The data confirm that the compounds are hydrophilic at all three ionic strengths studied (Table 1). There were, however, some slight variations in the experimentally determined log *D*_lip_ values at the different ionic strengths: the values from the LLE studies were in the range of −0.71 to −1.00 for epinephrine and −0.96 to −1.25 for norepinephrine.

**TABLE 1:** Log *D*_lip_ values at different buffer ionic strengths using 1% *m*/*v* ILE, determined by LLE [28] and EKC (this work).

			EKC log *D*_lip_		EKC log *D*_lip_	
	LLE (25°C) log *D*_lip_	LLE (25°C) log *D*_lip_	Epinephrine		Norepinephrine	
Buffer ionic strength (mM)	Epinephrine	Norepinephrine	25°C	37°C	25°C	37°C
**20**	−0.71	−1.22	0.35	0.45	0.49	0.43
**50**	−0.99	−1.25	−0.01	0.33	0.33	0.08
**100**	−1.00	−0.96	−0.42	0.25	0.11	0.17

In determining the distribution coefficients of epinephrine and norepinephrine by EKC, the experiments were conducted at the same three different ionic strengths as in the LLE studies. However, in addition to 25°C, also at 37°C. The *D*_lip_ values were calculated using Equations (1)–(5). The corresponding logarithmic values of the distribution coefficients, log *D*_lip_, of the compounds are given in Table 1. Some typical electropherograms are shown in Supporting Information (Figures S1–S4).

**FIGURE 2:**
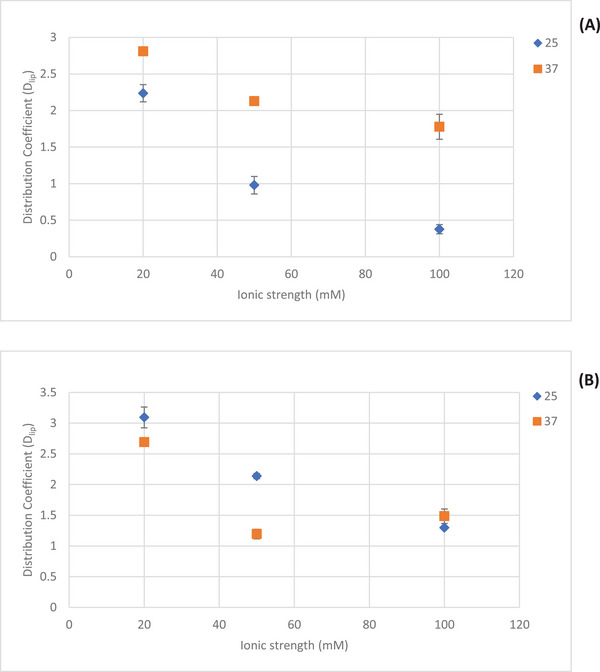
Distribution coefficients (*D*_lip_) of (A) epinephrine and (B) norepinephrine at different buffer ionic strengths at 25°C and 37°C using EKC with 1% (*m*/*v*) Intralipid as the pseudostationary phase (*n* = 3). Running conditions: BGE (CZE): PBS at pH 7.4 (*I* = 20, 50, 100 mM); BGE (EKC): 1 % (*m*/*v*) ILE at pH 7.4 (*I* = 20, 50, 100 mM); 60/68.5 cm effective/total length, 50/360 µm, ID/OD; temperature 25°C and 37°C; separation voltage +30 kV; sample injection: 100 mbar × 10 s; UV‐detection at 200 nm.

Overall, the log *D*_lip_ values determined by EKC were very close to zero and some values were even negative, implying that epinephrine and norepinephrine are considerably hydrophilic at all three tested ionic strengths (20, 50, and 100 mM) and both temperatures (25°C and 37°C) (Table 1). Irrespective of the temperature, both epinephrine and norepinephrine showed the highest distribution in ILE at 20 mM ionic strength. When the ionic strength was increased to 50 mM, and further to 100 mM, the distribution coefficient log *D*_lip_ slightly decreased for both epinephrine and norepinephrine, except for log *D*_lip_ of norepinephrine at 100 mM at 37°C that was higher than that at 50 mM ionic strength. Even though the distribution coefficient slightly changed from 50 to 100 mM ionic strengths, the log *D*_lip_ values were all quite closer to each other. When the temperature was increased from 25°C to 37°C, epinephrine and norepinephrine showed different responses. The distribution coefficient of epinephrine at 37°C was higher than at 25°C at all three ionic strengths, but for norepinephrine it was increased only at 100 mM ionic strength. At 20 and 50 mM ionic strength, the distribution coefficient of norepinephrine vaguely decreased with increasing temperature. Even though the log *D*_lip_ values were close to zero depicting the hydrophilic nature of the analytes, a clear variation in the distribution and the interactions can be observed with varying temperature and ionic strength. This can be seen even clearer in Figure 2, which shows the absolute values of the *D*_lip_ values. Even though the log *D*_lip_ values from the LLE experiments (at 25°C) and EKC differ slightly, they are all close to zero, which confirms that epinephrine and norepinephrine do not show significant distribution into the ILE.

The values and the variation of the calculated distribution coefficients *D*_lip_ at the above‐mentioned conditions are shown in Figure 2. The relative standard deviations (%RSD) of the *D*_lip_ values (*n* = 3) for norepinephrine at 25°C were 5.48, 2.00, and 1.23 at 20, 50, and 100 mM ionic strength, respectively. At 37°C, the corresponding values for the same compound were 2.10, 6.53, and 4.98. For epinephrine slightly higher %RSD variations were obtained; at 25°C the values were 5.28, 12.21, and 16.66 at 20, 50, and 100 mM ionic strength, respectively, and at 37°C the corresponding %RSD values were 1.00, 3.20, and 9.62.

**FIGURE 3:**
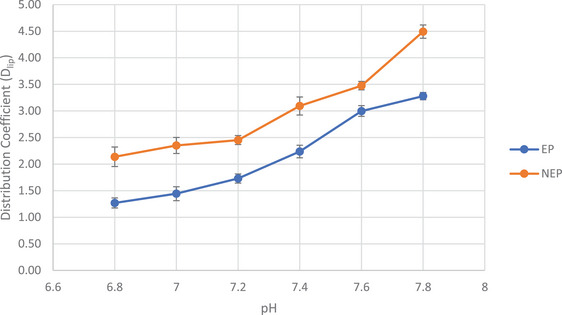
Distribution coefficients (*D*_lip_) of epinephrine and norepinephrine at different pH conditions using EKC with 1% (*m*/*v*) Intralipid as the pseudostationary phase (*n* = 3). Running conditions: BGE (CZE): PBS at pH 6.8–7.8 (*I* = 20 mM); BGE (EKC): 1 % ILE (*m*/*v*) at pH 6.8–7.8 (*I* = 20 mM); 60/68.5 cm effective/total length, (50/360 µm ID/OD); temperature 25°C; separation voltage +30 kV; sample injection: 100 mbar × 10 s; UV‐detection at 200 nm.

To investigate the impact of pH on the distribution coefficients, the study was conducted at six different pH values: 6.8, 7.0, 7.2, 7.4, 7.6, and 7.8. The pH range was selected based on the normal arterial blood pH, which falls within the range of 7.35–7.45. At lower pH values, physiological acidosis can occur. Acidosis refers to a state in the body where either the acid concentration has increased, there has been excessive loss of bicarbonate from the blood, or the kidneys and lungs are unable to eliminate sufficient acid from the body [32, 33]. For all these experiments, the ionic strength of the PBS and ILE was 20 mM and the running temperature was 25°C. Figure 3 illustrates the variations in the distribution coefficients *D* at the various pH values. An increase of the distribution coefficient *D* of both epinephrine and norepinephrine into the ILE with increasing pH was observed. This trend is inversely related with the degree of protonation of amino groups of the analytes: at higher pH values, the protonation is lower. Therefore, at higher pH values, the analytes were less charged, favoring higher degree of partitioning into the lipid phase than at lower pH value. This was reflected by the lower distribution coefficients *D* of the analytes at lower pH values compared to higher pH values.

### 3.2 Determination of the Binding Constants

**FIGURE 4:**
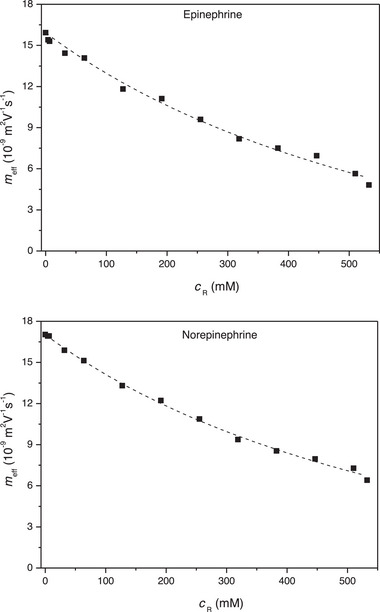
Nonlinear regression analysis of dependences of the effective mobility, *m*_eff_, of epinephrine and norepinephrine on the concentration, *c*_R_, of ILE in BGE. Running conditions: BGE: 1% ILE (*m*/*v*) at pH 7.4 (*I* = 20 mM); 60/68.5 cm effective/total length; 50/360 µm ID/OD; temperature 25°C; separation voltage +30 kV; sample injection: 100 mbar × 10 s; UV detection at 200 nm.

The dependences of effective mobilities of epinephrine and norepinephrine on the concentration of ILE in the BGE measured by EKC are shown in Figure 4. The effective mobilities of both analytes fit relatively well the curves modeling these dependences described by Equation 9. The nonlinear regression analysis of this data provided values of the binding constant, *K*_b_, and of the actual electrophoretic mobility, *µ*_AR_, of both analyte‐receptor (analyte‐ILE) complexes (Table 2). Good agreement between the experimental data and the theoretical model is also confirmed by the high values close to 1 of the adjusted *R*‐square coefficients of determination shown in Table 2. The binding constants of both analyte‐ILE complexes were very small, ca. 1.3 L mol^−1^, confirming very weak interactions between these drugs and ILE and their very low distribution in the ILE PSP. The effective mobilities of both analytes in the BGE free of ILE are very similar, being equal to 15.9 and 17.0 Tiselius unit (TU = 1 × 10^−9^ m^2^V^−1^), respectively. This is in accordance with approximately the same charge to size ratio of their molecules. The actual electrophoretic mobilities of the analyte‐ILE complexes differ in ca. 2 TU units. RSDs of the binding constants are in the acceptable range of 11.5%–21.3% as well as the RSDs of the electrophoretic mobilities of the complexes, 23.5%–36.5%.

**TABLE 2:** Studied compounds, their electrophoretic mobilities, *µ*_A_, in the BGE free of ILE, and the binding constants, *K*_b_, and the electrophoretic mobilities of their complexes with ILE receptor, *µ*_AR_, at 25°C. *R* ^2^‐adj., adjusted coefficient of determination.

Studied compounds	(*µ*_A_ ± SD)* × 10^9^ (m^2^V^−1^s^−1^)	RSD (%)	*K*_b_ ± SD (L mol^−1^)	RSD (%)	(*µ*_AR_ ± SD) × 10^9^ (m^2^V^−1^s^−1^)	RSD (%)	*R* ^2^‐adj.
Epinephrine	15.9 ± 0.07	0.5	1.27 ± 0.27	21.3	−10.4 ± 3.8	36.5	0.9915
Norepinephrine	17.0 ± 0.09	0.5	1.31 ± 0.15	11.5	−8.1 ± 1.9	23.5	0.9976

## 4 Conclusions

Interactions of epinephrine and norepinephrine with ILE were studied by CE and EKC methods. The distribution coefficients of these drugs in the ILE PSP and the binding constants of their complexes with ILE were extracted from the CZE and EKC data. The obtained binding constants showed very weak interactions between epinephrine and norepinephrine and ILE, suggesting only very low distribution of these drugs into the ILE PSP. Even though there are minor differences in the distribution coefficients at the two temperatures (25°C and 37°C), three ionic strengths (20, 50, and 100 mM), and six pH values (6.8, 7.0, 7.2, 7.4, 7.6, and 7.8) investigated, the study demonstrates that epinephrine and norepinephrine behave as considerably hydrophilic compounds under all these tested conditions. The results of this study show that the distribution of epinephrine and norepinephrine into the ILE decreases with decreasing pH (acidosis). Thus, there is a slightly decreased distribution of epinephrine and norepinephrine into the ILE under acidosis, frequently observed in patients undergoing ILE resuscitation. Thus, we conclude that ILE treatment for patients with acidosis due to cardiovascular collapse caused by drug toxicity may have a negligible impact on the cardiovascular effect produced by epinephrine or norepinephrine.

## Author Contributions

**Dumidu Perera**: investigation, methodology, formal analysis, visualization, writing–original draft, writing–review and editing. **Henri Ravald**: conceptualization, investigation, writing–original draft. **Veronika Šolínová**: investigation, formal analysis. **Václav Kašička**: conceptualization, supervision, funding acquisition, writing–original draft, writing–review and editing. **Ju‐Tae Sohn**: conceptualization, writing–review and editing. **Susanne K. Wiedmer**: conceptualization, funding acquisition, project administration, supervision, writing–original draft, writing–review and editing.

## Conflicts of Interest

The authors declare no conflicts of interest.

## Supporting information




**Supporting file**: jssc70188‐sup‐0001‐SuppMat.docx.

## Data Availability

Data available on request from the authors.
