# Supplementary material for: Interactions of Epinephrine and Norepinephrine with Intralipid Emulsion Studied by Capillary Electrokinetic Chromatography
Source: J Sep Sci. 2025 Jun 9;48(6):e70188. doi: 10.1002/jssc.70188 (PMC12146797; doi:10.1002/jssc.70188)
Supplement: Supplementary file 1 — Supporting file: jssc70188‐sup‐0001‐SuppMat.docx. [file JSSC-48-e70188-s001.docx]

Supplementary material

**Interactions of epinephrine and norepinephrine with Intralipid emulsion studied by capillary electrokinetic chromatography**

Dumidu Perera, Henri K. M. Ravald, Veronika Šolínová, Václav Kašička, Ju-Tae Sohn, Susanne K. Wiedmer

Figure captions

Figure S1. Separation of norepinephrine (100 µg/mL) using EKC with 1% (m/v) Intralipid as the pseudostationary phase (n=3). Running conditions: BGE (EKC): 1 % (m/v) ILE at pH 7.4 (I=50 mM); 60/68.5 cm effective/total length, 50/360 µm ID/OD; temperature 25 °C; separation voltage +30 kV; sample injection: 100 mbar × 10 s; UV-detection at 200 nm.

Figure S2. Separation of norepinephrine (100 µg/mL) using CZE with PBS (n=3). Running conditions: BGE (CZE): PBS at pH 7.4 (I=50 mM); 60/68.5 cm effective/total length, 50/360 µm ID/OD; temperature 25 °C; separation voltage +30 kV; sample injection: 100 mbar × 10 s; UV-detection at 200 nm.

Figure S3. Separation of epinephrine (100 µg/mL) using EKC with 1% (m/v) Intralipid as the pseudostationary phase (n=3). Running conditions: BGE (EKC): 1 % (m/v) ILE at pH 7.4 (I=20 mM); 60/68.5 cm effective/total length, 50/360 µm ID/OD; temperature 25 °C; separation voltage +30 kV; sample injection: 100 mbar × 10 s; UV-detection at 200 nm.

Figure S4. Separation of epinephrine (100 µg/mL) using CZE with PBS (n=3). Running conditions: BGE (CZE): PBS at pH 7.4 (I=20 mM); 60/68.5 cm effective/total length, 50/360 µm ID/OD; temperature 25 °C; separation voltage +30 kV; sample injection: 100 mbar × 10 s; UV-detection at 200 nm.


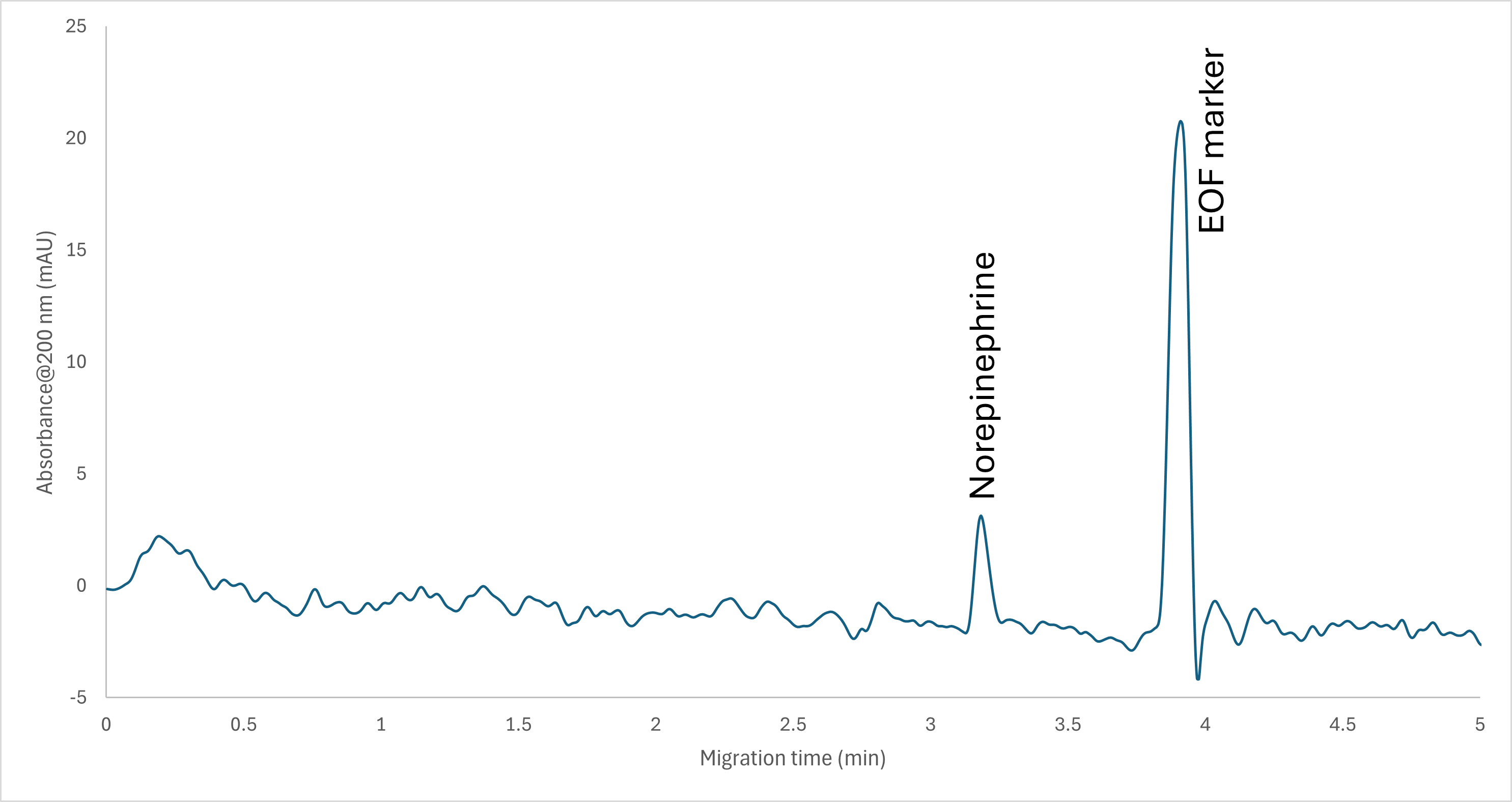


Figure S1. Separation of norepinephrine (100 µg/mL) using EKC with 1% (m/v) Intralipid as the pseudostationary phase (n=3). Running conditions: BGE (EKC): 1 % (m/v) ILE at pH 7.4 (I=50 mM); 60/68.5 cm effective/total length, 50/360 µm ID/OD; temperature 25 °C; separation voltage +30 kV; sample injection: 100 mbar × 10 s; UV-detection at 200 nm.


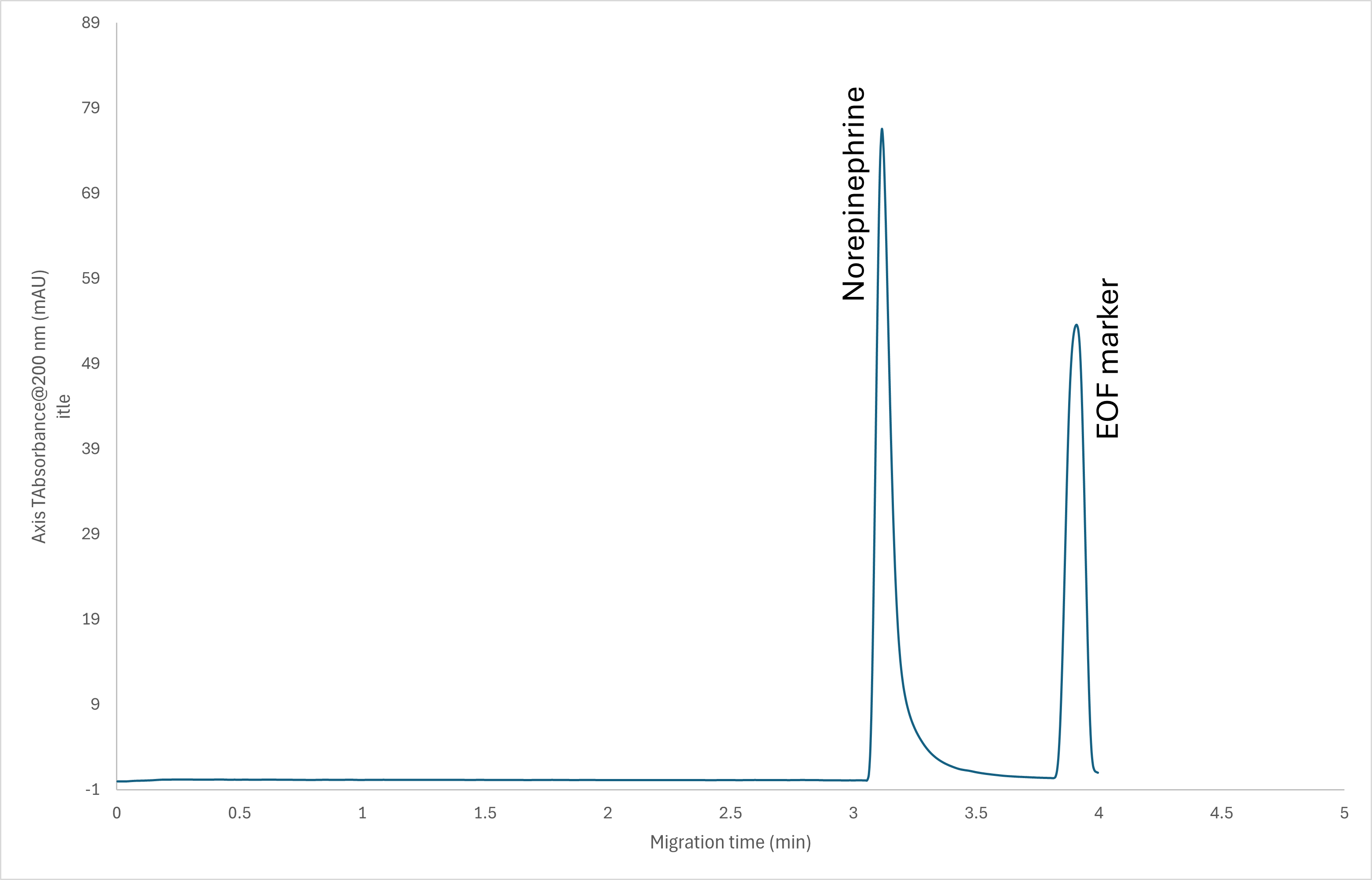


Figure S2. Separation of norepinephrine (100 µg/mL) using CZE with PBS (n=3). Running conditions: BGE (CZE): PBS at pH 7.4 (I=50 mM); 60/68.5 cm effective/total length, 50/360 µm ID/OD; temperature 25 °C and 37 °C; separation voltage +30 kV; sample injection: 100 mbar × 10 s; UV-detection at 200 nm.


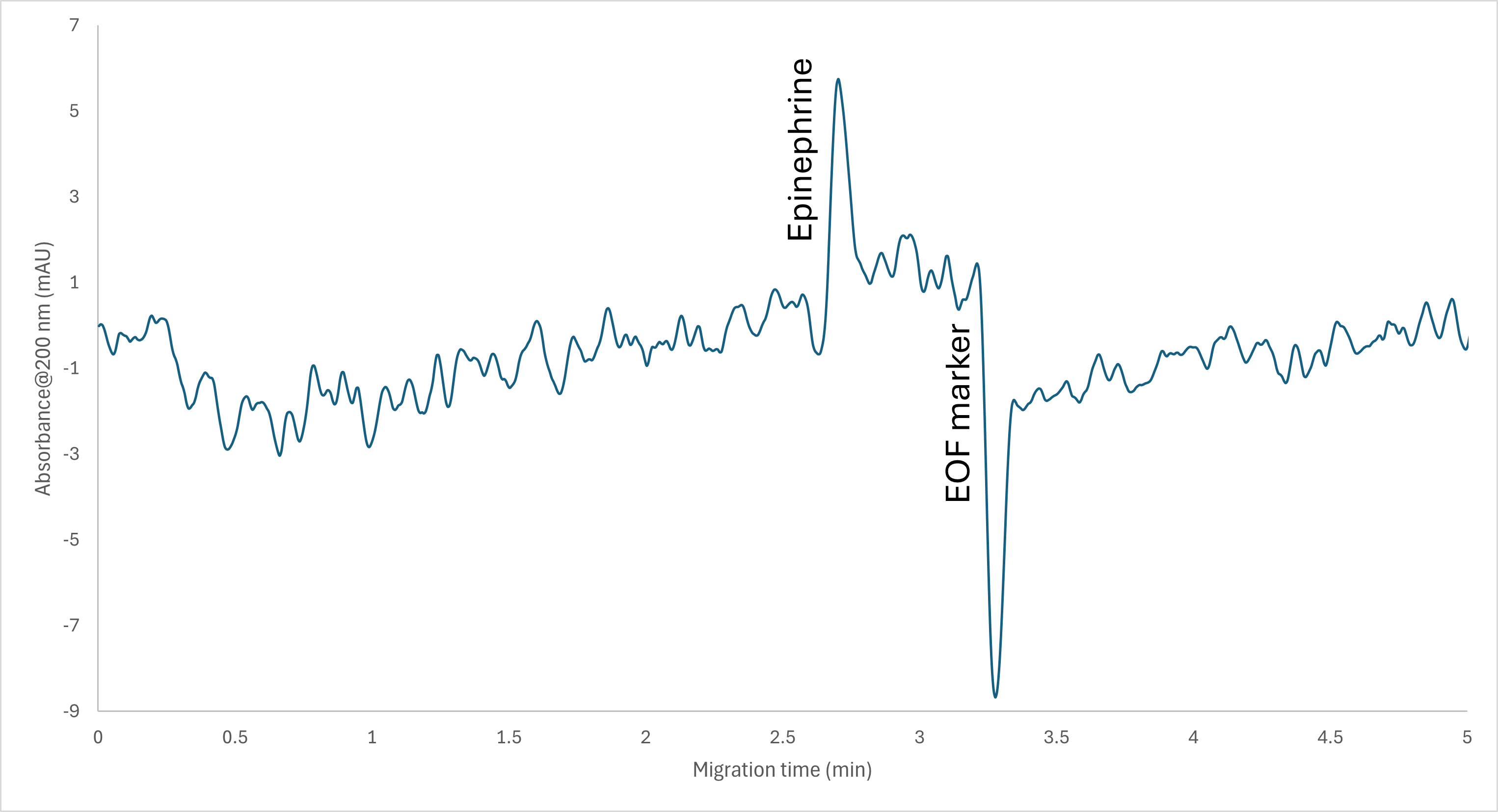


Figure S3. Separation of epinephrine (100 µg/mL) using EKC with 1% (m/v) Intralipid as the pseudostationary phase (n=3). Running conditions: BGE (EKC): 1 % (m/v) ILE at pH 7.4 (I=20 mM); 60/68.5 cm effective/total length, 50/360 µm ID/OD; temperature 25 °C; separation voltage +30 kV; sample injection: 100 mbar × 10 s; UV-detection at 200 nm.


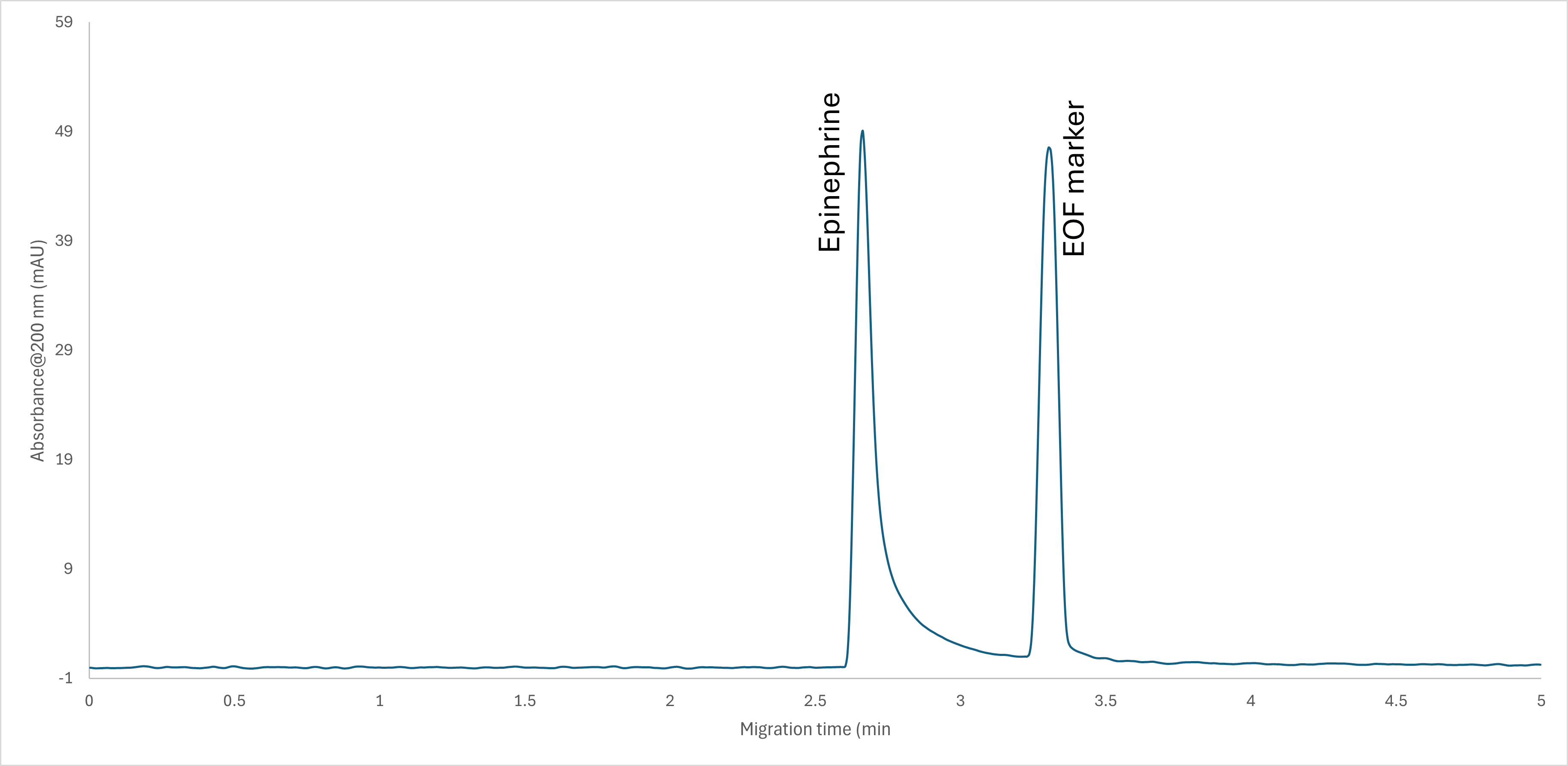


Figure S4. Separation of epinephrine (100 µg/mL) using CZE with PBS (n=3). Running conditions: BGE (CZE): PBS at pH 7.4 (I=20 mM); 60/68.5 cm effective/total length, 50/360 µm ID/OD; temperature 25 °C and 37 °C; separation voltage +30 kV; sample injection: 100 mbar × 10 s; UV-detection at 200 nm.
